# The complete chloroplast genome of *Callicarpa longifolia* Lamk. var. *floccosa* Schauer (Lamiaceae)

**DOI:** 10.1080/23802359.2021.2002210

**Published:** 2021-11-23

**Authors:** Chao-Ying Wang, Li-Hua Chen, Hui-Liu He, Yang Liu, Qian Wang

**Affiliations:** a Chongqing City Management College, Chongqing, China; b Chongqing Academic of Forestry, Chongqing, China; c Chongqing Key Laboratory of Plant Resource Conservation and Germplasm Innovation, Institute of Resources Botany, School of Life Sciences, Southwest University, Chongqing, China

**Keywords:** *Callicarpa longifolia* var. *floccose*, chloroplast genome, Lamiaceae, phylogenetic analysis

## Abstract

*Callicarpa longifolia* Lamk. var. *floccosa* Schauer is a species with medicinal and ornamental values. In this study, the complete chloroplast genome of *C. longifolia* var. *floccose* is reported. The chloroplast genome of this species is 154,285 bp in length and contains a typical circular quadripartite structure. There are two inverted repeats of 25,700 bp, which is separated by a large single-copy region of 85,008 bp and a small single-copy region of 17,877 bp. The complete chloroplast contains 112 distinct genes, including 78 protein-coding, 30 tRNAs and 4 rRNAs genes. Phylogenetic analysis suggests that *C. longifolia* var. *floccose* is closely associated with *C. formosana*.

*Callicarpa longifolia* Lamk. var. *floccosa* Schauer 1847 is a perennial shrub of genus *Callicarpa* and family Lamiaceae (Chen and Michael 1994, Li et al. 2016). It is mainly distributed in southwestern China, India, Singapore, Indonesia, and the Philippines. This species has high ornamental value with clustered white, purple or red berries, and also has certain medicinal value. In this study, the chloroplast (cp) genome characteristics of *C. longifolia* var. *floccose* were assembled and described for the first time. It will provide valuable resources for further study of genetic conservation, evolution, and molecular breeding studies in the genus of *Callicarpa* L.

Fresh leaves of *C. longifolia* var. *floccose* was collected from Pengshui County of Chongqing, China (N29°00′47.85″, E107°52′31.09″, 553 m). The voucher specimen (CY Wang PS 20191002) was deposited in the herbarium of Southwest University (SWCTU, Qian Wang, wangqian123@swu.edu.cn). Whole cp genome sequences were generated by using Illumina HiSeq 4000 platform at the Beijing Novogene Bioinformatics Technology Co., Ltd. (Nanjing, China). To obtain the complete cp genome, *de novo* assembly of the raw data was performed by using SPAdes version 3.5.0 (Bankevich et al. 2012). The cp genome sequence was annotated by using PGA (https://github.com/quxiaojian/PGA) (Qu et al. 2019) with manual adjustments. The sequence cp genome was deposited in GenBank (accession number MW149076).

The cp genome of *C. longifolia* var. *floccose* formed a typical circular quadripartite structure of 154,285 bp length, containing two inverted repeats (IRs) of 25,700 bp, a large single-copy (LSC) region of 85,008 bp, and a small single-copy (SSC) region of 17,877 bp. The GC content of the whole cp genome is 38.07%. In total, 112 distinct genes were annotated, including 78 protein-coding (PCGs), 30 transfer RNA (tRNA), and 4 ribosomal RNA (rRNA) genes. The gene content and organization are similar to other members of *Callicarpa* (Wang et al. 2019; Du et al. 2020; Wang et al. 2021; Xie et al. 2021).

To determine the phylogenetic position of *C. longifolia* var. *floccose* with related species, a phylogenetic analysis was performed based on 11 complete cp genomes of *Callicarpa*. In addition, *Dicrastylis parvifolia* (MT473755) was included as an outgroup. The sequences were aligned by MAFFT version 7.0 (Katoh and Standley 2013). The maximum-likelihood (ML) phylogenetic trees were reconstructed by using RAxML version 8.2.11 (Stamatakis 2014) with the GTR Gamma and 1,000 bootstrap replicates. As shown in the phylogenetic tree (Figure 1), *C. longifolia* var. *floccose* was most closely to *C. formosana*.

**Figure 1.**
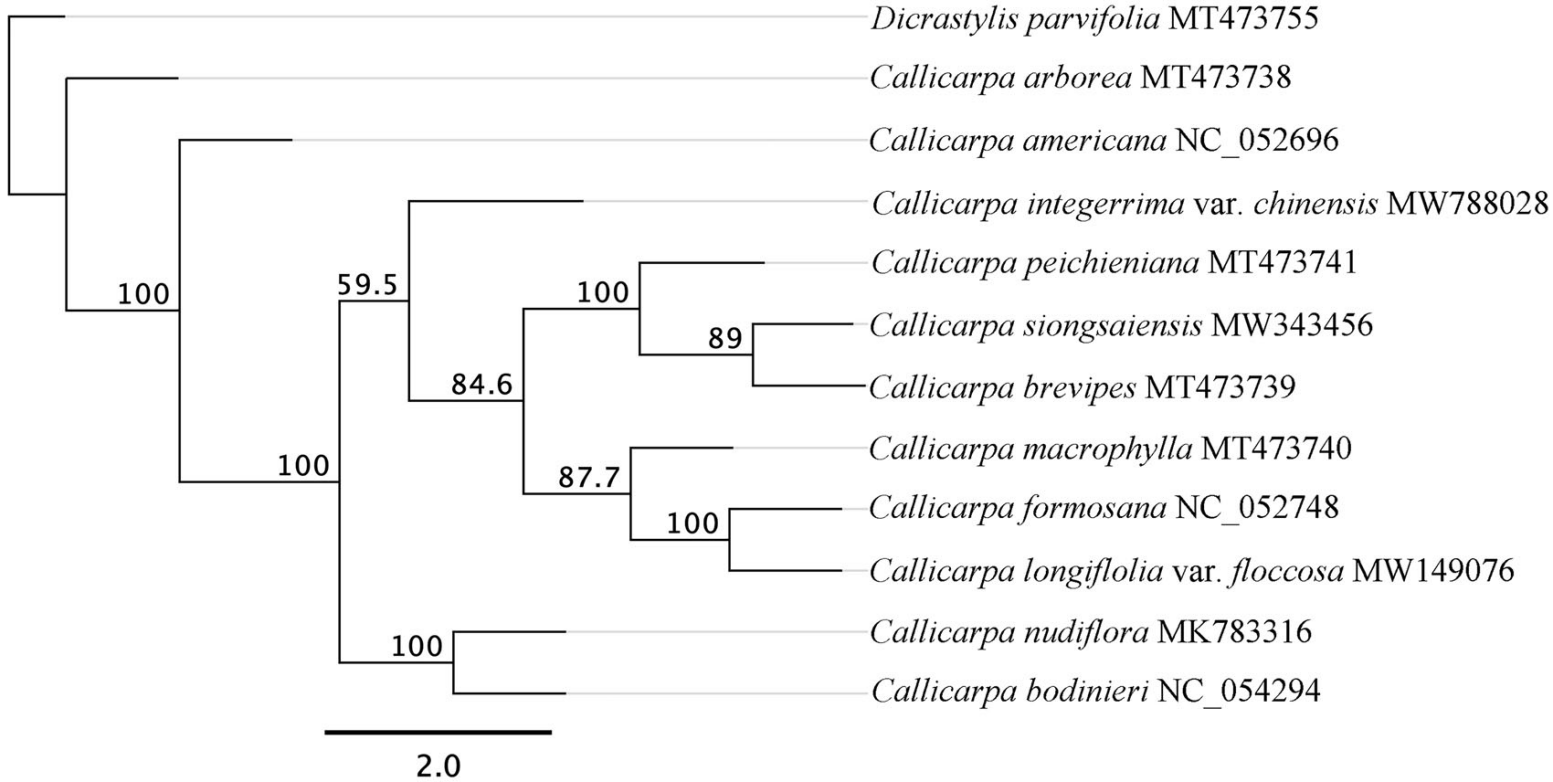
A maximum likelihood (ML) tree of genus Callicarpa based on twelve complete chloroplast genome sequences. Numbers at nodes correspond to ML bootstrap percentages (1,000 replicates). All the sequences are available in GenBank, with the accession numbers listed right to their scientific names.

## Data Availability

Chloroplast data supporting this study are openly available in GenBank at nucleotide database, https://www.ncbi.nlm.nih.gov/nuccore/MW149076, Associated BioProject, https://www.ncbi.nlm.nih.gov/bioproject/PRJNA757227, BioSample accession number at https://www.ncbi.nlm.nih.gov/biosample/SAMN20955945 and Sequence Read Archive at https://www.ncbi.nlm.nih.gov/sra/SRR15584543.
